# Underestimation of airway luminal eosinophilia by quantitative sputum cytometry

**DOI:** 10.1186/s13223-021-00567-w

**Published:** 2021-07-05

**Authors:** Melanie Kjarsgaard, Adil Adatia, Anurag Bhalla, Nicola LaVigne, Katherine Radford, Chynna Huang, Manali Mukherjee, Parameswaran Nair

**Affiliations:** 1 Firestone Institute for Respiratory Health, St. Joseph’s Healthcare, 50 Charlton Avenue East, Hamilton, ON L8N 4A6 Canada; 2 Department of Medicine, McMaster University, Firestone Institute for Respiratory Health, 50 Charlton Avenue East, Hamilton, ON L8N 4A6 Canada

**Keywords:** Eosinophils, Sputum cell counts, Th2 endotype

## Abstract

**Rationale:**

On Wright-stained sputum cytospins, eosinophil differential of ≥ 1.2% is considered abnormal, and ≥ 2.3% identifies an eosinophilic endotype. We hypothesized that failure to consider free eosinophil granules (FEG), and the re-emergence (unmasking) of eosinophilia due to various reasons underestimate the prevalence of the eosinophilic endotype.

**Methods:**

This is a retrospective analysis of our Institutional Review Board-approved clinical sputum database. Of the 24,176 examinations of sputa from patients with various airway diseases, 17,693 were viable cell counts from 9570 patients (6604 on a single occasion, 2967 from multiple occasions). The prevalence of intact eosinophil % at 1.2 and 2.3% thresholds was first examined. Then, additional evidence of eosinophilia was assessed by semi-quantitative enumeration of FEGs. In those patients whose sputa were examined on multiple occasions (at the time of an exacerbation or after corticosteroid dose was reduced), re-emergence (unmasking) of eosinophilia was assessed

.

**Results:**

Using the threshold of eosinophilia ≥ 1.2%, 6289/17693 (35.6%) of sputa were classified as eosinophilic. This increased to 7850/17693 (44.4%) when the presence of FEGs was considered. Using the threshold of eosinophilia ≥ 2.3%, 4647/17693 (26.3%) of sputa were classified as eosinophilic. This increased to 5435/17693 (30.7%) when the presence of FEG were considered. Extrapolating from the prevalence of re-emergence observed in the 2967 patients who had sputa examined on multiple occasions to the whole sample, we estimated that eosinophilia at 1.2% threshold would be observed in at least 60% of the samples, and a clinically relevant eosinophilia at 2.3% threshold would be observed in at least 48.5% of the samples.

**Conclusions:**

Using a large sputum cytometry clinical database (17,693 viable cell counts), we demonstrate that a single time point intact cell count underestimates the prevalence of eosinophilia in a variety of airway diseases. The prevalence of eosinophilia increases from 35.6 to 60% (40% underestimation) at the 1.2% threshold, and from 26.3 to 48.5% (45% underestimation) at the 2.3% clinically relevant threshold, when free granules and a second examination are considered. This has important implications to identify the eosinophilic and Th2 high endotype both for clinical trials of anti-eosinophil therapies, and to select patients who may respond well to glucocorticosteroids and anti-IL5 therapies.

## Introduction

Mucosal inflammation is a feature of airway diseases such as asthma, COPD, bronchiectasis, bronchiolitis, and chronic cough. This is an important contributor to airflow limitation. Luminal cellular inflammation can be assessed relatively non-invasively, reliably and reproducibly, by sputum cytometry. Quantitative cytometry on Wrights (or Giemsa) stained sputum cytospins provides total cellularity and white cell differential count [1]. The normal values have been established. For eosinophil differential %, the mean is 0.4%, and the upper limit of normality is 1.1% (90^th^ centile) or 2.2% (when 2 standard deviations are considered) [2]. For asthma, an eosinophil endotype is identified when the eosinophil % is greater than 2.2%, and a measurement > 3% is often considered clinically relevant [3]. However, these cut-offs take into consideration only intact eosinophils, and not degranulated eosinophils. Free eosinophil granules (FEG) in tissue is an indicator of eosinophilic activity [4]. In sputum, this correlates with eosinophil cationic proteins such as eosinophil peroxidase and the presence of moderate and many free granules correlate with clinical severity [5]. Additionally, eosinophil % may change over time when any associated neutrophilia may resolve [6], with exacerbations [7], or when the dosage of corticosteroids are reduced [8].

We hypothesized that the eosinophilic endotype may be underestimated using the current practices that may not use sputum cell counts at all or the examination be limited to a single time point intact cell count without considering FEGs. The objective of this study was to evaluate the prevalence of eosinophilia in sputum (using a cut-off of both 1.2% and 2.3%) in a large clinical database when free eosinophil granules, multiple sputum samples, and the effects of corticosteroids are taken into consideration. We did not examine differences between the different types of airway diseases or the severity of the diseases.

## Methods

This is a retrospective study of those patients who attended the Firestone Institute for Respiratory Health for assessment of sputum cell counts for any airway disease between July 2004 and September 2020. The results were entered into FileMaker Pro Version 16 (Claris International, Cupertino, CA), a clinical database maintained for this purpose. The database contained demographics, physician diagnosis, reason for the test, whether the patient was stable or exacerbated (clinical diagnosis), and dose of inhaled and oral corticosteroids. In addition, baseline and post-induction spirometry were also recorded. The sputum differential cell count parameters included total cell count (× 10^6^/g), and percentages of viable cells, squamous cell contamination, neutrophil, eosinophil, macrophage, lymphocyte, and bronchial epithelial cells. The presence of free eosinophil granules was enumerated by quantification of degranulated eosinophil clumps per field of view under 400 × magnification. They were recorded as none, 1–2 as few, 2–3 as moderate and > 3 as many. Sputum induction and examination of cell counts were performed as described by Pizzichini et al. [9]. Even if an intact cell differential count could not be made due to excessive degeneration, free eosinophil granules (FEG) were enumerated as previously described. Presence of moderate/many granules (not few granules) are regarded as clinically relevant to define an endotype [5].

To determine the prevalence of emerging eosinophilia, the dataset was separated into two groups, those patients with only one sputum cell count and those with two or more cell counts. Re-emergence of eosinophilia was defined as the absence of eosinophilia on an initial specimen and presence of eosinophilia on the subsequent specimen. Patients with several sputum measurements could have more than one episode of emergence. For example, emergence events could be seen between the first and second sputum samples and between the fourth and fifth samples within the same patient. Emergence of eosinophilia was assessed using two criteria: (1) eosinophil differential becoming ≥ 1.2% or few/moderate/many FEG identified on the subsequent sample or both and (2) eosinophil differential becoming ≥ 2.3% or moderate/many FEG identified on the subsequent sample or both. Each emergence event was categorized by suspected etiology. If the sputum neutrophil count was elevated in initial sample, the event was attributed to the treatment of bacterial bronchitis with antibiotic therapy, which could have unmasked airway eosinophilia. If the oral/inhaled corticosteroid dose was lower at the time of the subsequent sputum examination compared to the initial sample, the emergence event was attributed to a decrease in corticosteroid therapy. If neither of these conditions were present, the emergence event was attributed to a flare of eosinophilic inflammation.

Since re-emergence (unmasking) of eosinophilia was examined only in a small proportion of our patients, we extrapolated this fraction to the entire sample to estimate the prevalence in the whole sample. We validated this using a Markov chain model of uncertainty.

### Statistical analysis

Statistical analysis was performed using GraphPad Prism Version 8.3.1 (GraphPad Software, USA) and R 2020 (R Core Team, Austria). Parametric variables are presented as mean with standard deviation, non-parametric as median with minimum–maximum and categorical variables as percentage. In those patients with two or more cell counts, detailed database mapping assessed for events of emergent eosinophilia based on the predefined 1.2 and 2.3% thresholds on subsequent samples from the same patient. This was extrapolated to the whole sample (by multiplying by a factor of 28.1% for the 1.2% threshold and by multiplying by a factor of 25.6% for the 2.3% threshold). This was validated by an additional sensitivity analysis using a discrete time Markov chain model that estimated the probability that a patient’s sputum sample without eosinophilia would show eosinophilia on the next examination. Each sputum type (eosinophilic and non-eosinophilic) was conceptualized as a node and the transition probabilities in the Markov chain were estimated from the data using standard methodology.

## Results

There were 24,176 individual records that include both spontaneously expectorated and induced sputum. Of these, 6483 did not possess adequate sputum volume for analysis. The remaining 17,693 samples (obtained from 9571 patients) were analysed. The physician diagnoses for these samples were asthma (27%), COPD (21%), asthma + COPD (7%), chronic cough (20%), bronchiectasis (14%), other airway diseases and diffuse parenchymal diseases (11%). The demographic, clinical and sputum characteristics for patients who provided adequate sputum for analysis is shown in Table 1. 35.6% (n = 6289) samples had eosinophils > 1.2%, while 26.3% (n = 4647) samples had eosinophils > 2.3% based on just intact cell counts.

**Table 1:** Demographic, clinical and sputum characteristics for those patients whose sputum was viable for analysis (n = 17,693)

	Female, n (%)*	Age, years*	Daily dose ICS^†^ (mcg)*	On ICS, n (%)*	Daily dose OCS (mg)*	On OCS, n (%)*
	7296 (56.2)	58.0 (30.6)	637.9 (633.1)	13,298 (75.2)	3.4 (16.5)	5713 (32.3)

Viability	Differential cell count, 10^6^/g (%)^#^					
	Total cells	Neutrophil	Eosinophil	Macrophage	Lymphocyte	Bronchial epithelial
75.0 (0–100)	6.0 (0.1–398.1)	63.3 (0.3–100)	0.8 (0–97)	28.0 (0–99)	0.6 (0–26.7)	0.0 (0–52.0)

*ICS* Inhaled corticosteroids; ^†^Fluticasone equivalent; mcg microgram; *OCS* oral corticosteroids, *mg* milligram, *g* gram. *expressed as mean (standard deviation); #expressed as median (minimum–maximum)

### Eosinophilia when FEGs were considered

**Fig. 1:**
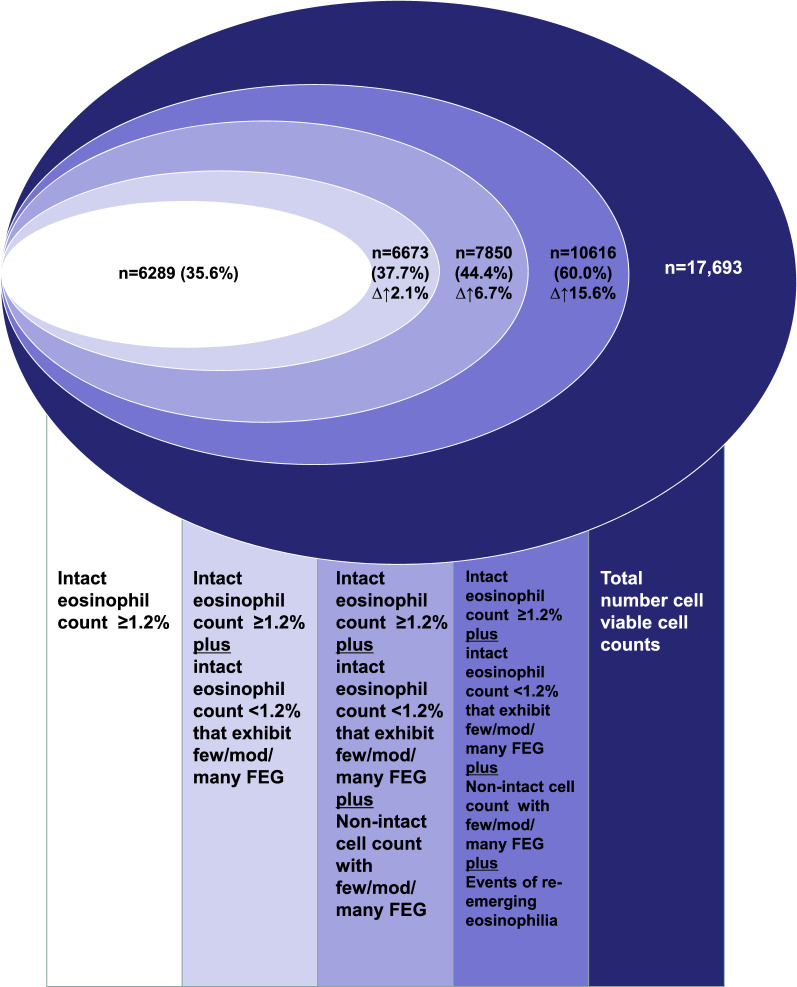
Sputum Eosinophilia at threshold of ≥ 1.2%. The Euler diagram show the incremental proportions when eosinophilia is assessed using a single intact cell differential, when free granules are considered, and when a second sample is examined (extrapolation to the whole sample from the subset who had re-emergence demonstrated)

**Fig. 2:**
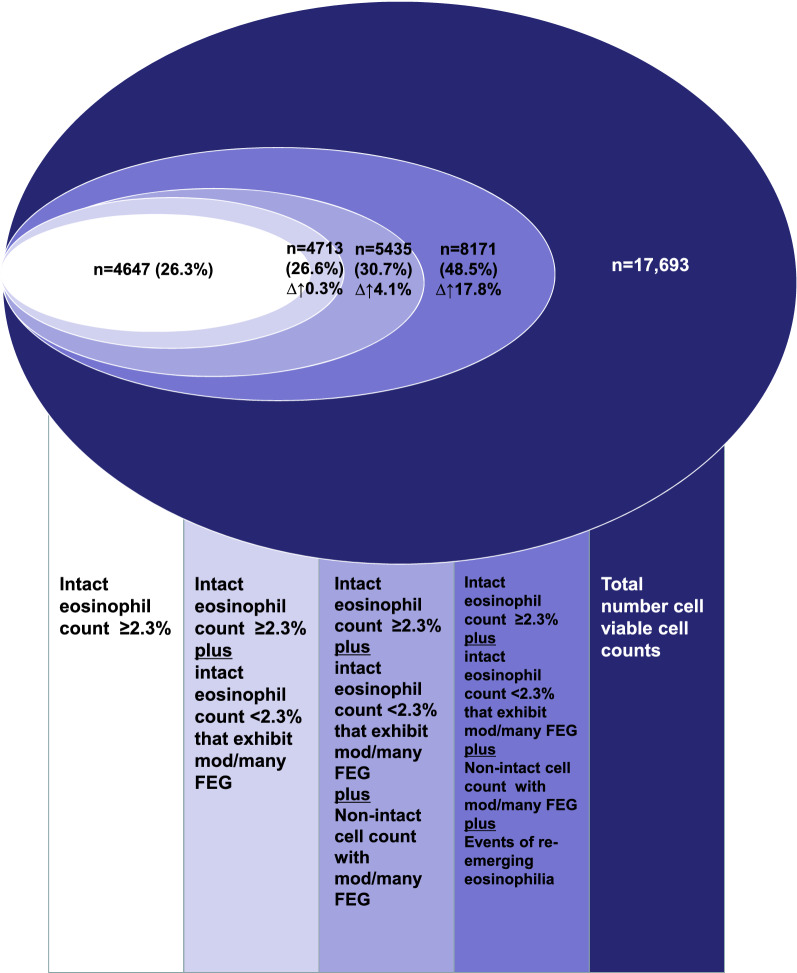
Sputum Eosinophilia at threshold of ≥ 2.3%. The Euler diagram show the incremental proportions when eosinophilia is assessed using a single intact cell differential, when free granules are considered, and when a second sample is examined (extrapolation to the whole sample from the subset who had re-emergence demonstrated)

At both the 1.2% and 2.3% thresholds, there were only a few additional samples with granules associated with intact cell differential (n = 384 and n = 66 respectively). But there were additional 1899 samples without intact cell differential but with FEGs that would have been mis-classified as non-eosinophilic. 1177 of these had few granules (likely not clinically relevant) and 722 had moderate or many granules (clinically relevant). Thus, when granules are taken into consideration, the prevalence of eosinophilia would increase at the 1.2% threshold from 35.6% to 44.4% (Fig. 1), and at the 2.3% threshold from 26.3 to 30.7% (Fig. 2).

### Eosinophilia when multiple samples were examined

There were 2967 patients who provided more than one sputum sample, and this subgroup of patients accounted for 11,088 of the sputum specimens. Using eosinophils ≥ 1.2% or any FEG, emergence of eosinophilia was seen in 834/28.1% of patients, and there were 0.385 events per patient. Using eosinophils ≥ 2.3% or moderate/many FEG, 759/25.6% patients had emergence of eosinophilia, and there were 0.365 events per patient. The suspected etiology for masked eosinophilia for each threshold his shown in Table 2. Disease flare was the most common purported reason for eosinophilia to be seen in a patient’s subsequent sputum sample regardless of threshold used.

**Table 2:** Suspected etiology of masked eosinophilia in those patients with two or more sputum cell counts (n = 2967)

Criteria for assessing eosinophilic inflammation	1.2%^¥^	2.3%^ǁ^
Treatment of neutrophilia and decrease in corticosteroid dose	9.2%	8.7%
Treatment of neutrophilia	19.1%	28.8%
Decrease in corticosteroid dose	26.5%	28.9%
Disease flare (not attributed to neutrophilia and/or decrease in corticosteroid dose)	57.8%	60.5%

^¥^Group defined as eosinophils ≥ 1.2% or any FEG

^ǁ^Group defined as eosinophils ≥ 2.3% or moderate/many FEG

We extrapolated this proportion of un-masking (re-emergence) of eosinophilia (28.1% at 1.2% threshold and 25.6% at the 2.3% threshold) to the whole population and estimated that an additional 15.6% samples at the 1.2% threshold would be classified as eosinophilic (Fig. 1) and an additional 17.8% samples at the 2.3% threshold would be classified as eosinophilic (Fig. 2).

Thus, we estimate that a total of 60% of our sputum examinations from our large clinical database of unselected respiratory diseases would be eosinophilic at the 1.2% threshold (Fig. 1) and 48.5% would be eosinophilic at the 2.3% threshold (Fig. 2). The respective numbers using the Markov model were comparable at 73 and 46% respectively.

## Discussion

**Fig. 3:**
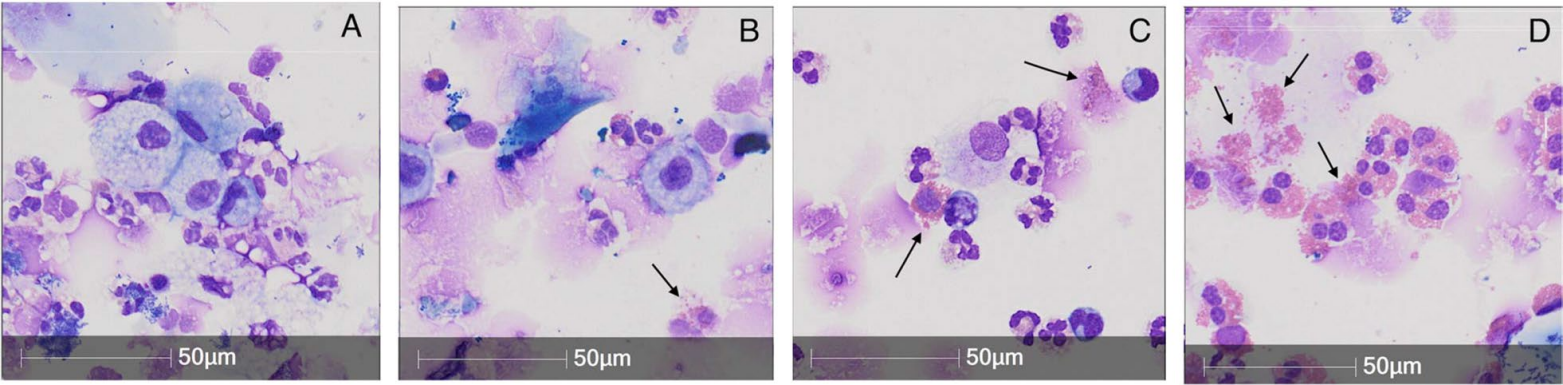
Microscopy images at 40 × magnification of sputum cytospins stained with Wright Giemsa demonstrating grading of free eosinophil granules (black arrows); **A** none, **B** few, **C** moderate and **D** many [12]

This retrospective study using a large clinical database demonstrates that eosinophilia in sputum may be underestimated by as much as 45% if conclusions are drawn from a single intact cell differential count. This emphasizes the importance of considering free eosinophil granules and to consider changes in eosinophilia associated with exacerbations, resolution of concomitant neutrophilia, or change in corticosteroid dosages. This has important clinical relevance, not only to endotype for selection of patients into clinical trials, but also to make therapeutic decisions about escalating or decrease corticosteroid dosage and initiation of eosinophil-specific biologic therapies (Fig. 3).

It is important, for number of reasons, to recognize free eosinophil granules in sputum. Eosinophil cytolysis and release of cationic proteins are a marker of severity and contribute to bronchial epithelial injury and impairs repair [10]. Eosinophil peroxidase contributes to the bromination of tyrosine residues [11] and is associated with epithelial dysfunction. Peroxidase activity also triggers an autoimmune reactive process in the airway [12] that has implications for disease severity and response to treatment of asthma with biologics [13]. Further, cytolysis and release of extracellular traps may lead to crystal formation within the airways [14, 15]. Indeed, recent evidence suggests that Charcot Leyden Crystals, that are the products of auto-crystallization of galectin-10 is regulated independent of IL-5 [16], and the failure to recognize this in sputum may further underestimate the eosinophilic activity in the airways. This needs further investigation. A further underestimation of airway eosinophilic activity may result from failure to recognize these granular proteins within airway macrophages [17].

A second important reason for underestimating airway eosinophilia is when conclusions are drawn from a single time point assessment. The three most clinically relevant factors that might affect eosinophil % are whether sputum was sampled during an exacerbation, the dose of corticosteroids at the time of sampling, and if there was a concomitant neutrophilia that may mask an underlying eosinophilia. Certain airway infections may also directly induce an eosinophilic response that may resolve over time [18]. Particularly in patients with COPD, neutrophilic and eosinophilic exacerbations may interchange over time [19].

A limitation of our study is its retrospective nature that is associated with all the inherent biases of such a study design. However, the strengths include the large sample size, robust laboratory methods, and regular stringent external quality control. Another limitation is that we did not characterize patients into specific disease categories such as asthma, COPD, overlap, bronchiectasis etc. This is because of the likely imprecision in the data coding based on a physician diagnosis. Given the large sample size, scrutiny of individual charts to confirm the physician diagnosis was not possible and we did not have approval from our Research Ethics Board for chart review. However, the purpose of this manuscript is not to relate eosinophilia to a particular type of airway disease or severity, rather to report fallacies in the estimation of eosinophilia in sputum analysis. Although our analysis was limited to assessment of eosinophilic activity, there might be other aspect of cellular inflammation that are often not taken into consideration leading to inaccurate characterization as a non-T2 endotype. These include lymphocyte [20] and mast cell numbers [21] in sputum that can be identified particularly with more advanced microscopy, flow cytometry, and mass cytometry. They could also be markers of corticosteroid responsiveness and it remains to be seen how often these endotypes may occur in the absence of eosinophilia. We estimate the extent of underestimation to be greater if we were to limit our analysis to patients with severe asthma. It is conceivable that there would be even greater misclassification into the eosinophil-low endotype of asthma using single time point estimation of low thresholds (eg 150 cells/µL) of blood eosinophil numbers.

In summary, we highlight the relevance of recognizing free eosinophil granules, and the limitation of drawing conclusions about the eosinophil endotype from a single time point intact sputum cell count. This is likely relevant to select patients for anti-eosinophil clinical trials, to interpret treatment responses, and to guide the use of corticosteroids and biologics to treat eosinophil-mediated airway diseases.

## Data Availability

The datasets used and/or analyzed during the current study are available from the corresponding author on reasonable request.
